# A comparison of the effects of acupoint injection combined with hyaluronic acid versus isolated hyaluronic acid for knee osteoarthritis: A protocol for systematic review and meta-analysis of randomized controlled trials

**DOI:** 10.1097/MD.0000000000023262

**Published:** 2020-11-20

**Authors:** Xing Zhou, Kemeng Xiang, Xiangyao Yuan, Zhenping Wang, Kuanglin Li

**Affiliations:** Taizhou Traditional Chinese Medicine Hospital, Zhejiang Province, China.

**Keywords:** acupoint injection, hyaluronic acid, knee osteoarthritis, protocol, randomized controlled trial, systematic review

## Abstract

**Background:**

Knee osteoarthritis (KOA) is a kind of degenerative osteoarthropathy, which causes joint pain and limited mobility, seriously affects the quality of life of the patient. Traditional Chinese Medicine acupuncture and moxibustion has been widely used to treat KOA, and acupoint injection is 1 of the acupuncture treatment methods. The purpose of this work is to evaluate the effectiveness of Acupoint injection combined with Hyaluronic Acid injection compared with isolated Hyaluronic Acid injection for KOA.

**Methods:**

We will search articles in 7 electronic databases including Chinese National Knowledge Infrastructure, Wanfang Data, Chinese Scientific Journals Database, Chinese databases SinoMed, PubMed, Embase, and Cochrane Library databases. All the publications, with no time restrictions, will be searched without any restriction of language and status, the time from the establishment of the database to October 2020. Two reviewers will independently assess the quality of the selected studies, NoteExpress and Excel software will be used to extract data, and the content will be stored in an electronic chart. Different researchers will separately screen the titles and abstracts of records acquired potential eligibility which comes from the electronic databases. Full-text screening and data extraction will be conducted afterward independently. Statistical analysis will be conducted using RevMan 5.4 software.

**Results:**

This study will evaluate the efficacy and safety of Acupoint injection combined with Hyaluronic Acid injection compared with isolated Hyaluronic Acid injection in the treatment of KOA, to provide high-quality, evidence-based clinical recommendations.

**Trial registration number:**

INPLASY2020100058

**Conclusion:**

This study will provide reliable evidence on whether Acupoint injection combined with Hyaluronic Acid injection compared with isolated Hyaluronic Acid injection is more effective in treating KOA.

## 1 Introduction

Knee osteoarthritis (KOA) is a kind of degenerative osteoarthropathy, which causes pain and functional limitations, and it is most prevalent in the elderly.^[1,2]^ In China, an epidemiological survey on KOA covering 17,459 samples showed that the prevalence of KOA was 1.5% for mild, 3.3% for moderate, and 3.9% for severe.^[3]^ KOA has caused a huge burden on the patients themselves, health services, and society.^[4]^ Therefore, it is particularly important to seek a good and effective conservative treatment for KOA, which can improve the symptoms of osteoarthritis, delay the development of KOA, and reduce the possibility of knee replacement.

The pathological mechanism of KOA is currently unclear, but it is related to factors such as previous trauma, inflammation, and metabolic disorders.^[5]^

A variety of inflammatory cells in the synovial fluid of osteoarthritis, including cytokines (TNF, IL1β, IL6), growth factors (TGFβ, VEGF,), prostaglandins (PGE2), etc, which can induce matrix metalloproteinases and other hydrolases enzymes, that cause cartilage destruction.^[6,7,8]^ Eventually, cause joint dysfunction and joint pain eventually.^[9]^

Early to mid-stage KOA patients often receive non-steroidal anti-inflammatory drugs drug treatment,^[10]^ or intra-articular injection of Hyaluronic Acid(HA). HA may have an anti-inflammatory effect, can nourish cartilage and lubricate joints, and can partially relieve the symptoms of KOA patients.^[11]^ Although HA is no longer recommended in the guidelines of the American Academy of Orthopaedic Surgeons(AAOS), it is due to its exact clinical effect, many surgeons are still using it.^[12,13,14]^

Traditional Chinese Medicine (TCM) acupuncture and moxibustion plays an increasingly important role in the treatment of KOA and has achieved good results.^[15,16,17]^ Acupoint injection is a treatment method derived from TCM acupuncture and modern injection techniques have also had a profound impact on it. Acupoint injection is the direct injection of drugs on acupoints, which combines acupuncture stimulation, drug properties, and penetration of acupoints. Studies have shown that acupoint injection has a good curative effect in the treatment of KOA, with little side effects.^[18,19]^ Due to the pain source of KOA not only comes from the synovial, joint Capsules, and subchondral bone in the joint cavity, but also the fascia, ligament, and tendon attachment points outside the joint cavity.^[5,20]^ Considering this situation, some surgeons use an intra-articular injection of HA combined with acupoint injection to treat KOA, eager to solve the problem of pain in the knee joint cavity and outside the knee joint cavity. but still lacks a large sample of multi-center supported by evidence from clinical randomized controlled trials (RCT). Therefore, this work aims to achieve the above-mentioned goals through systematic reviews and meta-analysis, and provide reliable evidence for the clinic.

## 2 Methods

### 2.1 Study registration

This protocol report is structured according to the Preferred Reporting Items for Systematic Reviews and Meta-analysis Protocols statement.^[21]^ It is registered on the International Prospective Register of Systematic Reviews. (Registration number INPLASY2020100058; https://inplasy.com/inplasy-2020-10-0058/.)

### 2.2 Inclusion criteria

#### 2.2.1 Type of study

Only RCT will be included irrespective of blinding, publication status, or language in this study.

#### 2.2.2 Types of participants

Patients were diagnosed with KOA and the study belongs to a randomized controlled trial. Clinical results included Western Ontario and McMaster Universities Arthritis Index scores, Lequesne index score, Lysholm score, Japanese Orthopaedic Association score, clinical effectiveness, and visual analog scale. The experimental group must cover Acupoint injection combined with HA injection.and the control group must cover intra-articular injection with HA. Otherwise, studies will be excluded if they cannot meet the inclusion criteria.

#### 2.2.3 Types of Interventions

The intervention of the experimental group must cover Acupoint injection combined with HA injection. There are no restrictions on the way of dosage and treatment period.

#### 2.2.4 Types of control groups

The control group must cover the intra-articular injection with HA.

#### 2.2.5 Outcomes.

##### 2.2.5.1 Primary outcome measures

The primary outcome is Western Ontario and McMaster Universities Arthritis Index scores scores.^[22]^

##### 2.2.5.2 Secondary outcomes

The secondary outcome is clinical effectiveness, Lequesne index score,^[23]^ Lysholm score,^[24]^ Japanese Orthopaedic Association score score,^[25]^ visual analog scale,^[26]^ and the incidence of adverse reaction.

### 2.3 Search strategy

Chinese National Knowledge Infrastructure, Wanfang, Chinese Scientific Journals Database, Chinese databases, PubMed, Embase, and Cochrane Library databases were searched for this study. Take the subject terms combined with free words to search, take PubMed as an example: terms consist of disease (osteoarthritis, knee OR KOA OR knee osteoarthritides OR Osteoarthritis of Knee OR Osteoarthritis of the Knee) and intervention (Acupoint Injection OR Acupuncture Point Injection OR point injection therapy OR Acupuncture Points)and Comparison

(Sodium Hyaluronate OR Hyaluronic acid) and research types (randomized controlled trial OR controlled clinical trial OR random trials). as shown in Table 1.

**Table 1:** pubmed database search strategy.

Search number	Items
1	“Osteoarthritis, Knee”[Mesh]
2	osteoarthritis, knee[Title/Abstract]
3	knee osteoarthritis[Title/Abstract]
4	knee osteoarthritides[Title/Abstract]
5	Osteoarthritis of Knee[Title/Abstract]
6	Osteoarthritis of the Knee[Title/Abstract]
7	1 OR 2 OR 3 OR 4 OR 5 OR 6
8	Acupoint Injection[Title/Abstract]
9	Acupuncture Point Injection[Title/Abstract]
10	point injection therapy[Title/Abstract]
11	Acupuncture Points[Title/Abstract]
12	8 OR 9 OR 10 OR 11
13	Hyaluronic acid[Title/Abstract]
14	Sodium Hyaluronate[Title/Abstract]
15	13 OR 14
16	randomized controlled trial[Title/Abstract]
17	controlled clinical trial[Title/Abstract]
18	random trials[Title/Abstract]
19	16 OR 17 OR 18
16	7 AND 12 AND 15 AND 19

### 2.4 Data collection and analysis

#### 2.4.1 Selection of studies

**Figure 1:**
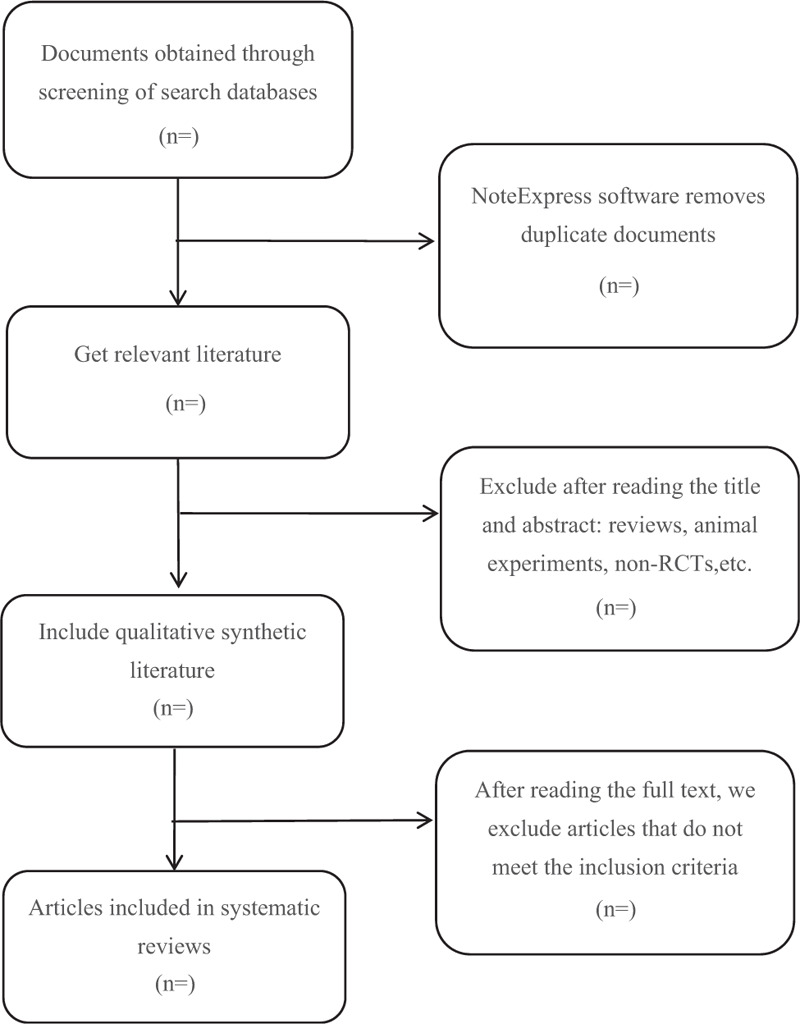
Flowchart of literature selection.

Different researchers will separately screen the titles and abstracts of records acquired potential eligibility which comes from the electronic databases. The obtained literature is managed by Notoexpress, irrelevant and duplicate articles are excluded by reading the title and abstract, Full texts screening and data extraction will be conducted afterward independently, and finally selected according to the inclusion criteria, Any disagreement will be resolved by discussion until consensus is reached or by consulting a third author. Preferred Reporting Items for Systematic Reviews and Meta-analysis Protocols flowchart Will be used to show the selection procedure (Fig. 1).

#### 2.4.2 Data extraction and management

NoteExpress and Excel software will be used to extract data, and the content will be stored in an electronic chart. The following data will be extracted: author, year of publication, country, interventions of experimental groups and control groups, time point, outcome measures, age of patients, the total number of people included in the study, patients’ basic information, etc. Different researchers will separately extract data. Any disagreement regarding data extraction will be resolved by discussion until consensus is reached or by consulting a third author.

### 2.5 Risk of bias assessment

Two reviewers will independently assess the quality of the selected studies according to the Cochrane Collaboration's tool for RCT.^[27]^ Items will be evaluated in 3 categories: Low risk of bias, unclear bias, and high risk of bias. The following characteristics will be evaluated: random sequence generation (selection bias), allocation concealment (selection bias), blinding of participants and personnel (performance bias), incomplete outcome data (attrition bias), selective reporting (reporting bias), and other biases. Results from these questions will be graphed and assessed using Review Manager 5.4. The results will be presented in the form of a graph and will be independently evaluated by 2 researchers. If there are differences of opinion, they will be discussed with the third researcher

### 2.6 Statistical analysis

Statistical analysis will be conducted using RevMan 5.4 software. For continuous data, will be used mean difference as the effect indicator with 95% confidence interval, and dichotomous data will be calculated as risk ratio or odds ratio as the effect index with 95% confidence interval. The *I*^2^ statistic will be used to assess levels of the heterogeneity, when *I*^2^<50%, the fixed-effect model can be used for analysis, otherwise, the random-effect model will be used.

### 2.7 Sensitivity analysis

Through sensitivity analysis assess the source of heterogeneity, by excluding low-quality studies, or by excluding 1 of the included studies in turn, if there is no significant change in the heterogeneity, the results are robust, otherwise, the excluded study may be the heterogeneous originate.

### 2.8 Subgroup analysis

We will consider the subgroup analysis intervention of the experimental group.

### 2.9 Publication bias

In this study, less than ten RCT will use funnel plots to evaluate publication bias, or else, Egger regression test will be used.

## 3 Discussion

Acupoint injection is derived from TCM acupuncture and moxibustion. It plays the dual role of acupuncture point stimulation and the efficacy of the injected drugs. It has achieved good results in the clinical treatment of KOA and is safe and reliable therapy. The purpose of using acupoint injection combined with HA is to solve more pain sources of KOA. However, there is still a lack of higher-level evidence-based medicine evidence to support this choice. Therefore, this study is to provide a more credible basis for future clinicians to make decisions.

This study still has certain shortcomings, because some factors will lead to biased results, such as low-quality original research, the intervention period, etc, which will weaken the reliability of the evidence.

## Author contributions

**Conceptualization:** Xing Zhou, Kemeng Xiang.

**Data curation:** Xing Zhou, Kemeng Xiang, Xiangyao Yuan, Zhenping Wang, Kuanglin Li.

**Formal analysis:** Xiangyao Yuan, Kuanglin Li.

**Investigation:** Zhenping Wang, Kuanglin Li.

**Methodology:** Xing Zhou, Kemeng Xiang, Kuanglin Li.

**Software:** Kemeng Xiang, Zhenping Wang.

**Supervision:** Xiangyao Yuan, Zhenping Wang, Kuanglin Li.

**Writing – original draft:** Xing Zhou, Kemeng Xiang.

**Writing – review & editing:** Xiangyao Yuan, Zhenping Wang.
